# A Study of Clinico-Microbiological Profile and Treatment Outcomes of Infectious Keratitis

**DOI:** 10.7759/cureus.71160

**Published:** 2024-10-09

**Authors:** Abhay Lune, Supriya Pokle, Ozukhil Radhakrishnan, Swaranjali Gore, Naina Chaturvedi

**Affiliations:** 1 Ophthalmology, Dr. D.Y. Patil Medical College, Hospital and Research Centre, Pimpri, IND; 2 Ophthalmology, Cornea, Glaucoma, Dr. D.Y. Patil Medical College, Hospital and Research Centre, Pimpri, IND; 3 Ophthalmology, Pediatric Ophthalmology and Neurophthalmology, Dr. D.Y. Patil Medical College, Hospital and Research Centre, Pimpri, IND

**Keywords:** clinico-microbiological profile, india, infectious keratitis, study, treatment outcomes

## Abstract

Background

Infectious keratitis (IK) is one of the major causes of corneal blindness in developing and developed nations. Numerous infections, including bacterial, fungal, viral, and protozoa, have been linked to IK. Corneal perforations can happen as one of the complications of IK. In this study, we aim to determine the clinical and microbiological profile and to study the treatment outcomes of IK.

Material and methods

It is a prospective, observational, and experimental study conducted in a tertiary care hospital and research center in Western Maharashtra, India, from September 2022 to June 2024. The sample size was 107 patients with IK. All patients were above 15 years of age, and all cases of microbial keratitis were within the inclusion criteria. Patients less than 15 years of age, patients with any other associated corneal pathology, healed cases of infectious keratitis, peripheral ulcerative keratitis, patients not willing to participate in the study, or not giving consent were excluded. Patients were examined thoroughly, and corneal scraping samples were collected and sent for microbiological examination. Appropriate treatment was provided through medical or surgical management, and treatment outcomes were evaluated during frequent follow-up visits.

Results

In our study, the majority of patients originated from rural areas, with most being over 60 years of age. Diabetes mellitus was seen in 24.3%, and a history of ocular trauma was present in 48.5% of patients. *Staphylococcus epidermidis* (3.74%) was the most common organism seen on gram staining, followed by *Staphylococcus aureus* (2.8%). *Pseudomonas aeruginosa* was the only gram-negative organism seen. The number of patients who had fungal isolates positive on culture was 57%, 12.1% had bacterial, and 30% had no growth of microorganisms. *Fusarium* species were seen in 39.2% of culture isolates, followed by *Aspergillus flavus* and *Niger* in 7.47% each. In 96% of medically managed patients, ulcers healed within two months. All ulcers with severe and moderate depth required therapeutic penetrating keratoplasty. A graft was in place in 95.2% after therapeutic penetrating keratoplasty, while one patient had graft rejection, and in one patient, endophthalmitis was seen.

Conclusion

*Staphylococcus epidermidis* was the most common bacterial agent isolated on gram staining, and *Pseudomonas aeruginosa* was the only gram-negative bacillus isolated on gram staining. Fungal isolates were the most common isolates observed on culture, seen in more than half of the IK patients in our study. The majority of patients who underwent medical management had ulcers healed in two months. The majority of the patients who underwent surgical management had fungal isolates positive. We recommend further research to be conducted in a generalized population, including urban settings, to understand the risk factors, microbiological profile, and treatment outcomes for IK.

## Introduction

Infectious keratitis (IK) is one of the essential contributors to corneal blindness in industrialized and impoverished nations [1]. IK, sometimes referred to as a “corneal ulcer”, is a condition that causes vision impairment that may be associated with several insults, including trauma, infection, degeneration, and inflammation of the corneal layers [2]. IK is a potentially vision-threatening eye disorder. It is generally associated with sudden eye discomfort, reduced vision, corneal ulceration, or infiltration of the corneal stroma [2]. Corneal blindness accounts for 5% of all worldwide blindness occurrences and is the third most common cause after cataracts and glaucoma [3]. Furthermore, it is estimated that corneal opacity and ulcers lead to approximately 1.5 to 2.0 million instances of corneal blindness in one eye annually, indicating a significant and unaddressed impact on human health [4].

Numerous infections, including bacterial, fungal, viral, and protozoa, have been linked to IK. Furthermore, studies show that polymicrobial infection is responsible for roughly 2-15% of all instances of IK [5-8]. Along with *Herpes* simplex virus type 1, other common causes of IK include bacterial infections, such as *Staphylococcus aureus*, *Streptococcus pneumonia*, *Pseudomonas aeruginosa* (Pa), and coagulase-negative *Staphylococci*. Infectious keratitis can also result from fungal infections; common culprits include *Aspergillus fumigatus*, *Fusarium* species, *Penicillium* species, *Candida albicans*, and *Fusarium flavus*. In addition, the illness may be brought on by parasites like *Acanthamoeba* [9]. 

Bacterial keratitis (BK) is a very prevalent and potentially serious ocular infectious condition that poses a significant risk to vision [5]. A bacterial infection poses a significant risk to the avascular corneal stroma, and patients may have unfavorable clinical results if immediate and effective treatment is not administered. Corneal perforations have been reported in the presence of highly aggressive bacteria, including *Pseudomonas aeruginosa* and *Staphylococcus aureus*; these perforations can occur within a day [10].

The study aims to understand the clinical and microbiological profile of IK patients and to ascertain their treatment outcomes after giving appropriate medical or surgical management.

## Materials and methods

Study design

This was a prospective observational and experimental study conducted in the Department of Ophthalmology at a tertiary hospital and research center in Western Maharashtra over 18 months from September 2022 to June 2024.

Sample size

After considering the prevalence of bacterial causes in IK in the parent study (49.3). Taking a confidence interval of 95%, with an acceptable difference of 10% and loss to follow-up to 10%, the calculated sample size was 107 patients with IK.

Ethics and consent 

The study received approval from the Institutional Ethics Committee of Dr. D. Y. Patil Medical College And Hospital, Pune, (approval number: IESC/PGS/2022/115) on September 28, 2022. Before beginning the study, informed written consent was obtained from each patient. 

Inclusion criteria

All patients were above 15 years of age, and all had cases of microbial keratitis.

Exclusion criteria

Patients less than 15 years of age, patients with any other associated corneal pathology, healed cases of infectious keratitis, peripheral ulcerative keratitis patients, non-compliant patients, or patients not willing to participate in the study.

Assessment of patients with IK

A detailed ophthalmic examination of each patient was performed, and a comprehensive history of each patient was taken, which includes the demographic profile, history of present complaints, duration of onset of symptoms, history of associated risk factors such as ocular trauma, contact lens use, use of topical steroid eyedrops, and history of associated systemic conditions such as diabetes mellitus (DM).

Patient's ocular evaluation

Visual acuity was measured for distant vision in every patient through visual acuity charts. A detailed ocular adnexal examination was conducted on each patient to examine structures such as eyelids, eyelashes, eyebrows, and lacrimal drainage pathways. A slit lamp biomicroscopic examination of the anterior segment was performed to assess corneal ulcer defect size, depth, and location and to examine the conjunctiva, anterior chamber, iris, pupil, and lens.

Corneal scraping samples were collected from the corneal ulcer using a sterile 15D blade after instilling anesthetic eyedrops in the affected eye. The scraping material was placed on two sterile glass slides and was inoculated on sterile culture media such as blood agar, chocolate agar, and Sabouraud dextrose agar. Gram staining, ZN staining, KOH mount and culture, and sensitivity were performed for the samples in a microbiology laboratory for isolating causative microorganisms. Appropriate medical management was given, and in severe cases, surgical management with therapeutic penetrating keratoplasty (TPK) was performed. Regular follow-ups with short intervals were done, and treatment outcomes were assessed.

Statistical analysis

Data was entered using MS Excel and analyzed with IBM Corp. Released 2019. IBM SPSS Statistics for Windows, Version 26.0. Armonk, NY: IBM Corp. Frequencies and proportions were estimated for categorical data. The mean (SD) and median (IQR) were estimated for continuous variables. Appropriate bar graphs and pie charts were created to display the results.

## Results

Table 1 shows the mean age of the patients in the present study was 57.71 years. The majority of the patients were above 60 years (42.1%), while 35.5% and 21.5% were in the age group 45-60 years and 25-44 years, respectively.

**Table 1:** Age distribution of patients present in the study population

Age distribution	No.	Percentage
15–24 years	1	0.9
25–44 years	23	21.5
45–60 years	38	35.5
>60 years	45	42.1
Total	107	100.0

**Figure 1:**
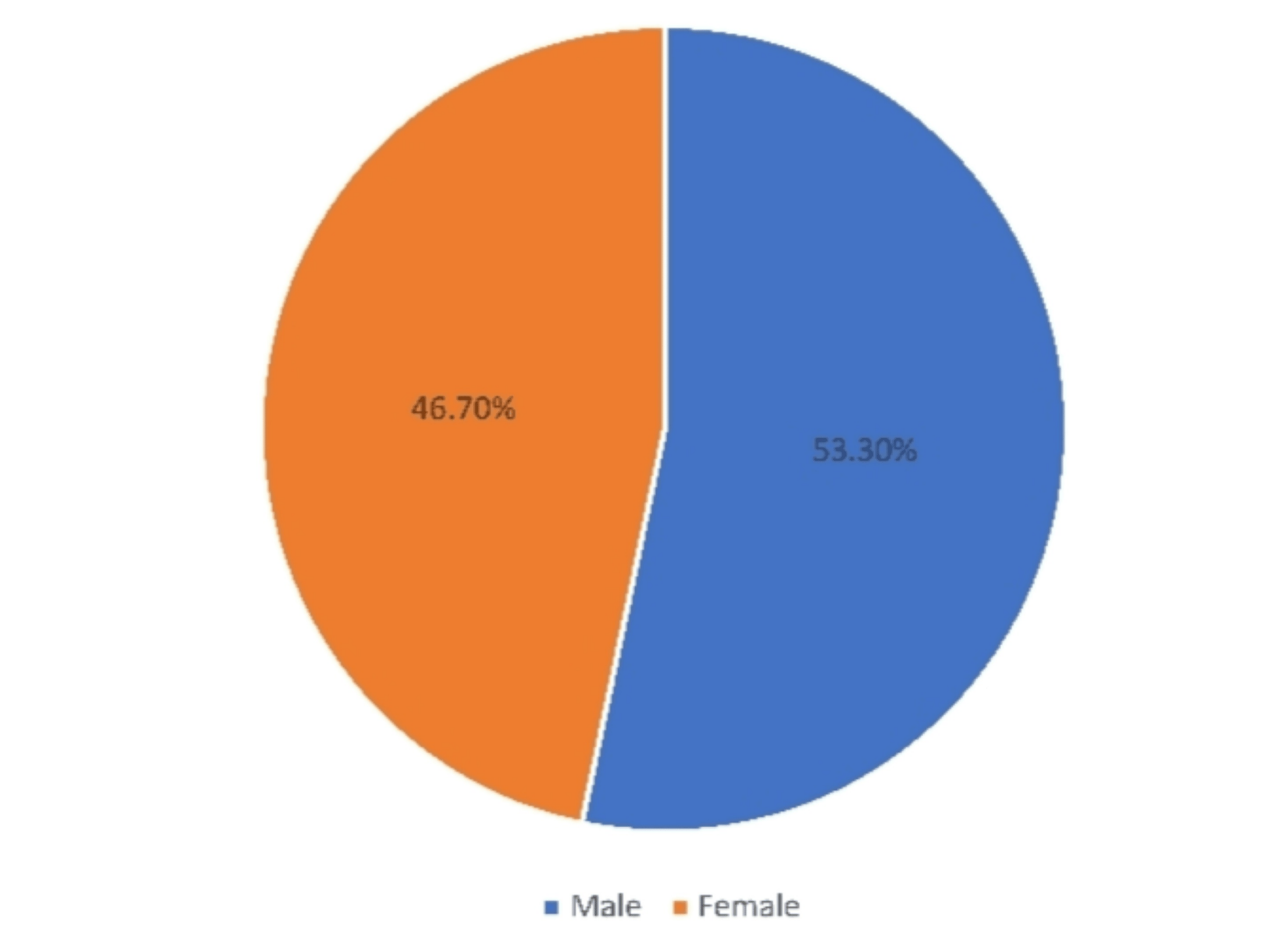
Pie chart showing sex distribution of patients in the study population Out of 107 patients in the study, 57 (53.3%) were male and 50 (46.7%) patients were female.

Figure 1 demonstrates that the majority of patients in our study-57- were male (53.3%), while 50 (46.7%) patients were female.

**Figure 2:**
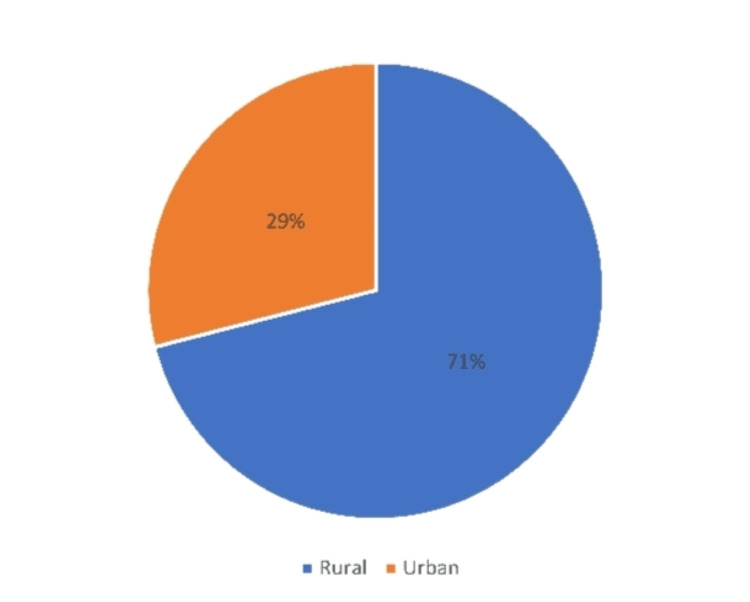
Pie chart showing distribution of patients based on whether the patient is from a rural or urban area Out of total 107 patients, 76 (71%) belonged to rural area and 31 (29%) patients were from urban area.

Figure 2 shows that the majority of the patients-76-belonged to rural areas (71%), while 31 (29%) were from urban areas.

**Figure 3:**
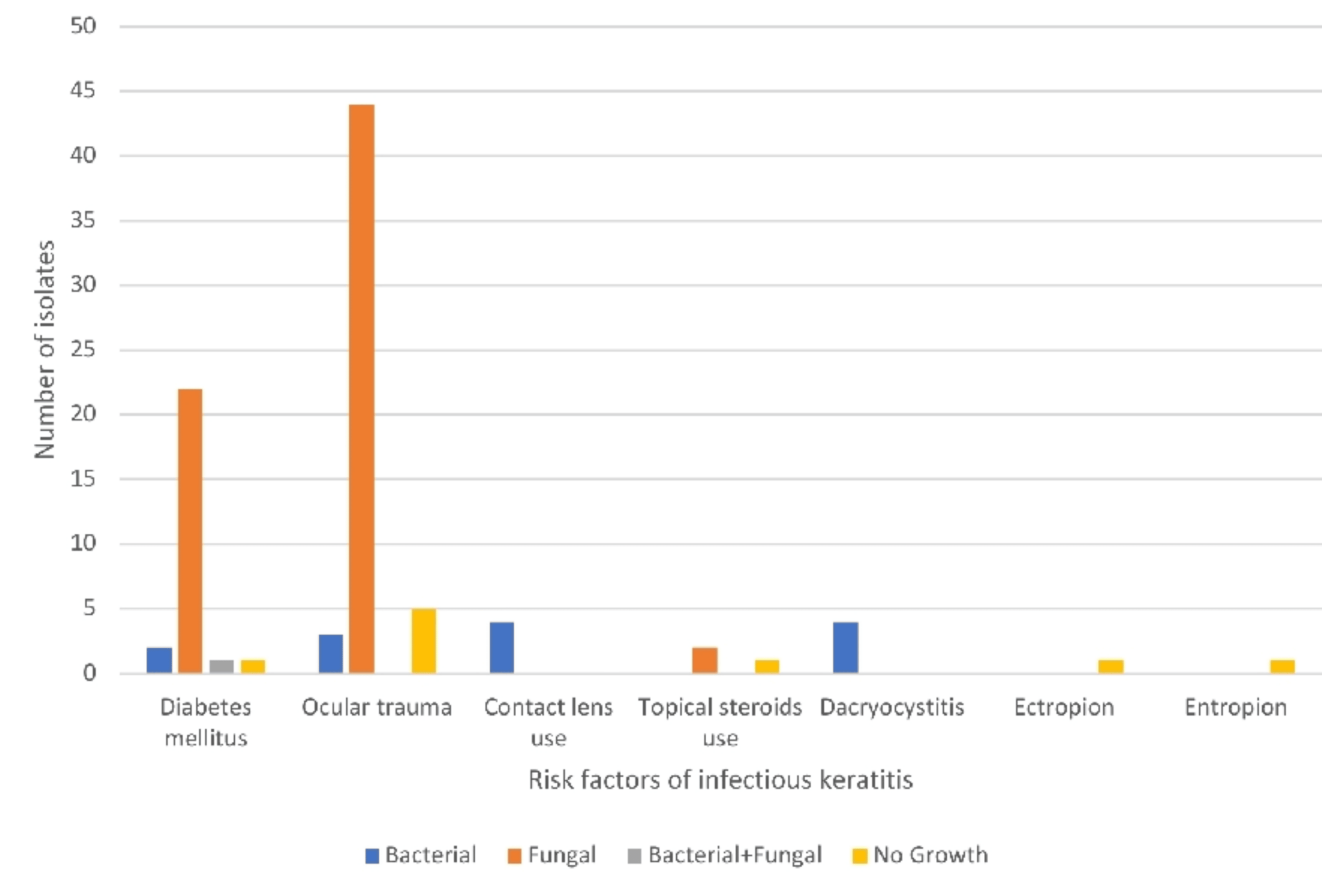
Predisposing risk factors of infectious keratitis in the study population

Tables 2, 3, and Figure 3 show ocular trauma was the most common risk factor seen in 48.5% of patients, and ocular trauma with vegetative matter was the most common type of trauma seen in most of them. A history of DM was present in 24.3% of patients, followed by a history of contact lens use, and dacryocystitis in 3.73% of patients, respectively. History of topical steroid use was seen in 2.8% of people. In patients with a history of DM, the majority of them had fungal isolates positive (84.61%). Ocular trauma with vegetative matter was the most common type of trauma. Most of the patients with a history of ocular trauma had fungal isolates positive (84.61%).

**Table 2:** Predisposing risk factors in infectious keratitis in the study population

	No. (out of 107)	Percentage
Ocular trauma	52	48.5
Diabetes mellitus	26	24.3
Contact lens use	4	3.73
Dacryocystitis	4	3.73
Topical steroids use	3	2.8
Ectropion	1	0.9
Entropion	1	0.9

**Table 3:** Predisposing risk factors for infectious keratitis in the study population

	Total	Bacterial	Fungal	Bacterial + Fungal Both	No growth
Ocular trauma	52 (100%)	3 (5.77%)	44 (84.6%)	0 (0.0%)	0 (0.0%)
Diabetes mellitus	26 (100%)	2 (7.69%)	22 (84.6%)	1 (3.85%)	1 (3.85%)
Contact lens use	4 (100%)	4 (100%)	0 (0.0%)	0 (0.0%)	0 (0.0%)
Dacryocystitis	4 (100%)	4 (100%)	0 (0.0%)	0 (0.0%)	0 (0.0%)
Topical steroids use	3 (100%)	0 (0.0%)	2 (66.6%)	0 (0.0%)	1 (33.33%)
Ectropion	1 (100%)	0 (0.00%)	0 (0.00%)	0 (0.00%)	1 (100%)
Entropion	1 (100%)	0 (0.00%)	0 (0.00%)	0 (0.00%)	1 (100%)

Table 4 shows the majority of the patients had a moderate-size ulcer (41.1%) followed by a severe-size ulcer (40.2%). The majority of patients had a mild depth ulcer (54.2%) followed by moderate depth (24.3%) and severe depth ulcer (21.5%).

**Table 4:** Clinical features of ulcer at presentation in the patients

Clinical features of ulcer	No.	Percentage
Size (Ulcer defect)		
Mild (< 2mm)	20	18.7
Moderate (2–5 mm)	44	41.1
Severe (> 5mm)	43	40.2
Depth (% Corneal thickness)		
Mild (<20%)	58	54.2
Moderate (20–50%)	26	24.3
Severe (>50%)	23	21.5

Table 5 demonstrated that *Staphylococcus epidermidis* was the most common gram-positive cocci isolated (3.74%), followed by *Staphylococcus aureus* (2.8%). *Pseudomonas aeruginosa* was the only gram-negative bacteria isolated (1.86%).

**Table 5:** Microorganisms isolated on gram staining

Microorganisms isolated on gram staining	No.	Percentage
GRAM POSITIVE COCCI		
Staphylococcus epidermidis	4	3.74
Staphylococcus aureus	3	2.8
Streptococcus pyogenes	2	1.86
Streptococcus pneumoniae	2	1.86
Streptococcus viridans	1	0.9
GRAM NEGATIVE BACILLI		
Pseudomonas aeruginosa	2	1.86
NEGATIVE RESULTS	93	87
TOTAL	107	100

ZN staining was negative in all patients (107) in the study. In 58% of patients, fungal elements were isolated on KOH mount.

**Figure 4:**
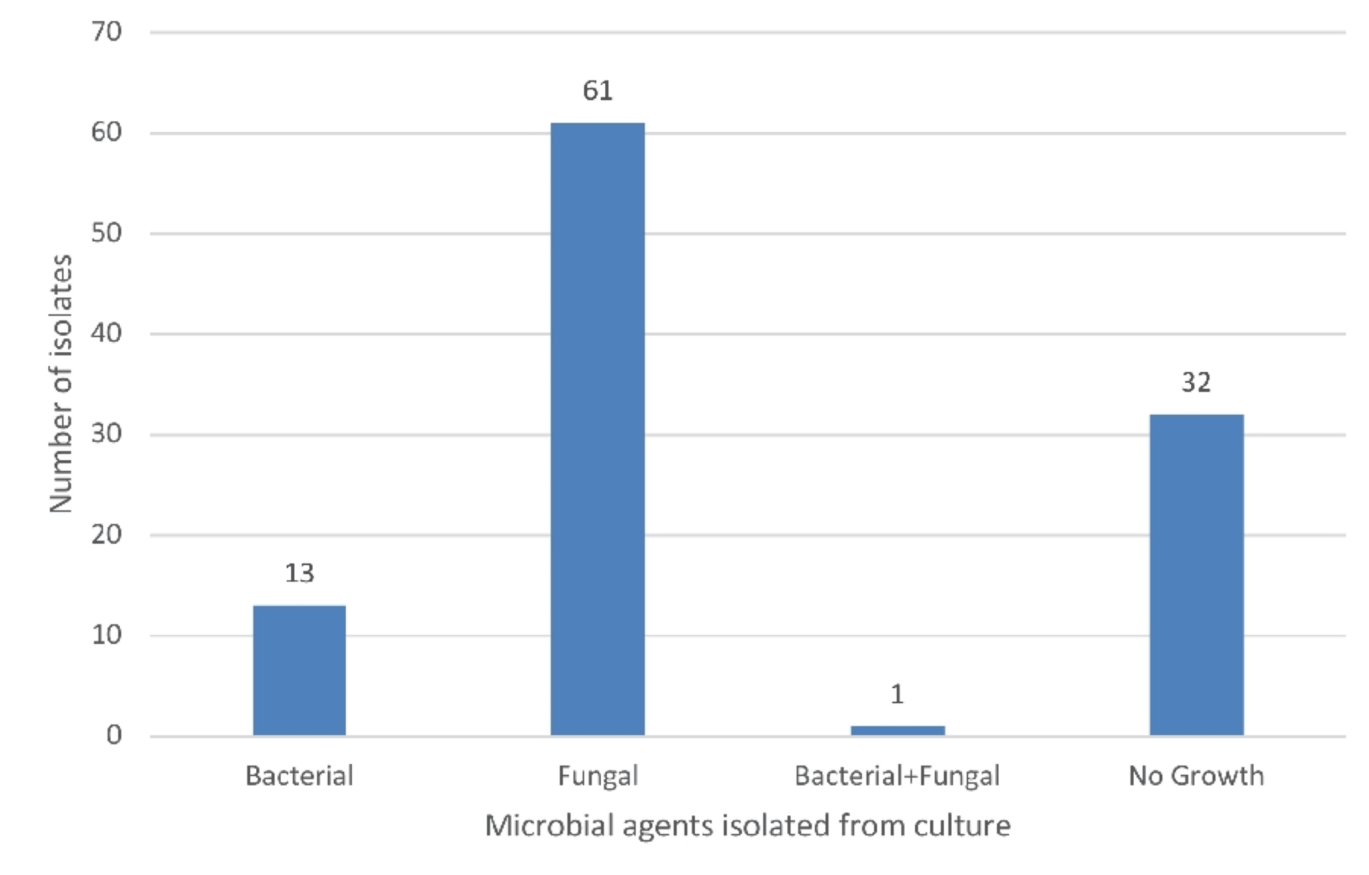
Bar diagram showing microbial agents isolated from culture Out of 107 patients, 61 had fungal isolates, no growth was seen in 32 patients, and 13 patients had bacterial isolates on culture. In one patient, both bacterial and fungal isolates were seen.

Figure 4 shows fungal organisms were the most common isolates in the culture (57%), followed by no growth in 30% and bacterial growth in 12.1%. In one patient, both bacterial and fungal isolates were positive.

Table 6 shows, on culture, that among the bacterial isolates, *Staphylococcus epidermidis* (3.7%) was the most common. Among the fungal isolates, *Fusarium* species (39.2%) were the most common organism. In one patient, both bacterial and fungal isolates of *Staphylococcus aureus* and *Fusarium* species were present.

**Table 6:** Microbial agents isolated on culture in patients with infectious keratitis

Microbial agents isolated on culture	No.	Percentage
Bacterial Isolates		
Pseudomonas aeruginosa	2	1.9
Staphylococcus aureus	2	1.9
Staphylococcus epidermidis	4	3.7
Streptococcus pyogenes	2	1.9
Streptococcus pneumonia	2	1.9
Streptococcus viridans	1	0.9
Fungal isolates		
Aspergillus flavus	8	7.47
Aspergillus fumigatus	3	2.8
Aspergillus niger	8	7.47
*Fusarium* species	42	39.2
Bacterial + Fungal	1	0.9
No growth	32	30

The total number of patients: 107. Patients lost to follow-up: 14 (13%). Patients for whom treatment outcomes were calculated: 93 (107-14).

Table 7 indicates that among the patients receiving medical management, 41.2% had positive fungal isolates, followed by patients with no growth and bacterial isolates at 39.20% and 19.60%, respectively. In the majority of patients who were treated with surgical management (TPK), fungal isolates were positive (83.3%).

**Table 7:** Type of management done for infectious keratitis patients

Management	Total	Bacterial	Fungal	Bacterial+ Fungal	No growth
Medical	51 (100.0%)	10 (19.6%)	21(41.2%)	0 (0.0%)	20 (39.2%)
Surgical (TPK)	42 (100.0%)	1 (2.4%)	35 (83.3%)	1 (2.4%)	5 (11.9%)

Table 8 depicts that the ulcer was completely healed with medical management in most of the patients (96%) by the two-month follow-up visit. The ulcer size was reduced but not completely healed in two patients (4%) by the two-month follow-up visit.

**Table 8:** Ulcer completely healed or not in patients with medical management

Medical management	No	Percentage
Ulcer completely healed (By two months)	49	96
Ulcer not healed (By two months)	2	4
Total	51	100

Table 9 shows a total of 51 patients with medical management, where, in the majority of the patients, the ulcer was healed with nebulomacular opacity (72.5%) or with leucomatous opacity (23.5%). One patient was seen with descemetocele, and in one patient, a non-healing ulcer was present. The patient with descemetocele was advised to undergo a corneal patch graft, and in the non-healing ulcer patient, neutrophic component was noted and was managed with lubricating drops.

**Table 9:** Treatment outcomes in patients with medical management

Medical management treatment outcomes	No.	Percentage
Ulcer healed with nebulomacular opacity	37	72.5
Ulcer healed with leucomatous opacity	12	23.5
Descemetocele	1	2
Ulcer not healed	1	2
Total	51	100

Table 10 shows that out of a total of 11 mild-sized ulcers, 54.5% of ulcers were healed by the 15-day follow-up and 45.5% were healed by the one-month follow-up. Out of a total of 37 moderate-sized ulcers, 32.4% were healed by the 15-day and two-month follow-up. And 29.8% of ulcers were healed by the one-month follow-up. Out of three severe-sized ulcers, 66.66% were healed by two months, and one (33.33%) ulcer was healed by the 15-day follow-up. One ulcer was not healed, and in one ulcer, a descemetocele was noted.

**Table 10:** Comparison between ulcer size and time required to heal the ulcer

Ulcer size	No. (%)	Healed by 15 days	Healed by 1 month	Healed by 2 months
Mild	11 (100%)	6 (54.5%)	5 (45.5%)	0 (0.00%)
Moderate	37 (100%)	12 (32.4%)	11 (29.8%)	12 (32.4%)
Severe	3 (100%)	1 (33.33%)	0 (0.00%)	2 (66.66%)

**Figure 5:**
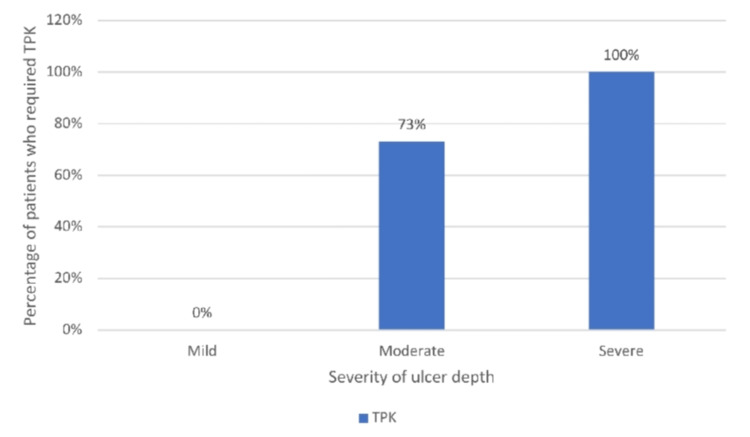
Bar diagram showing comparison between ulcer depth and TPK surgery performed In 100% severe and 73% moderate ulcer depth patients, therapeutic penetrating keratoplasty (TPK) was done.

Figure 5 shows that in all (100%) severe-depth ulcer patients, TPK was done. In 73% of moderate-depth ulcer patients, TPK was done.

**Figure 6:**
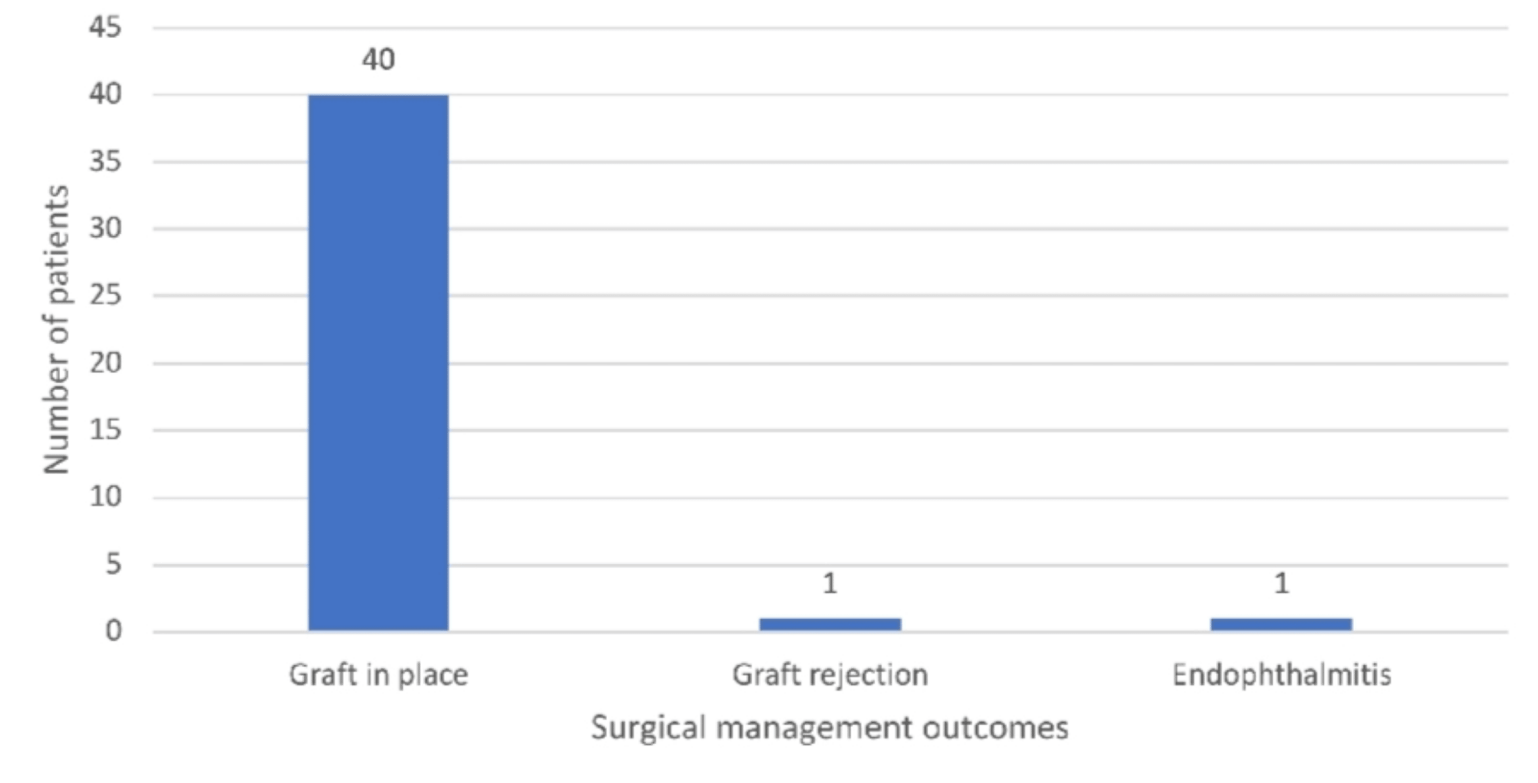
Bar diagram showing treatment outcomes in patients with surgical management Out of 42 surgically managed patients, 40 had grafts in place; one patient had endophthalmitis, and one patient had graft rejection.

Figure 6 shows that among the 42 patients managed surgically, most of the patients had a graft in place (95.2%), while 2.4% of patients had complications such as graft rejection, and 2.4% of patients had endophthalmitis.

## Discussion

We conducted a prospective, observational, and experimental study to assess the microbiological agent profile in IK. In addition, we determined the prevalence of various microorganisms responsible for the condition, and our study found the risk factors and clinical features associated with IK and treatment outcomes.

In our study, the average age of the patients (in years) was 57.71. Most patients were above 60 years (42.1%), while 35.5% and 21.5% were in the age group 45-60 years and 25-44 years. In the Ting et al. [5] study, among 1333 with IK enrolled, the average age (in years) was 49.9 ± 22.2. Similarly, Green et al. [11] documented IK in 3182 patients; the average age (in years) was 53 ± 22.6. Over half of the study patients were male (53.3%) in our study. In the study conducted by Ting et al. [5] and Green et al. [11], the proportion of females was in the range of 45 to 50. This distribution of gender was similar to our study. 

Around 71% of patients in our study were from rural settings. Similar observations were observed in Asian countries, which are probably limited to referral tertiary care access, leading to delayed presentation with complications [12-14]. Additionally, patients who have used traditional eye drugs documented delayed presentation and lower visual acuity in the long term [15]. 

In our study, 24.3% had diabetes. The majority (84.6%) of diabetic patients had fungal isolates positive. We observed 48.5% of ocular trauma history, and ocular trauma with vegetative matter was the most common etiology overall. For patients with a history of ocular trauma, the majority (84.6%) of them had fungal isolates positive. Among the cases, 3.73% used contact lenses, and 2.8% were using steroids. Dacryocystitis (3.73%) was the most common ocular adnexal abnormality. In our study, about half had ocular trauma as a risk factor, and ocular trauma with vegetative matter was the most common, probably because of the inclusion of the rural population undergoing agricultural occupation, while in developed countries, contact lens usage was the higher risk factor.

In the Indian setting, a study by Kumar et al. [14] revealed trauma (78.5%) as the primary risk factor. Similar observations were made by Ganguly et al. [16] and Dhakhwa et al. [17] in Nepal, probably because of the inclusion of agricultural rural populations. The findings were observed similarly in the neighboring country, Pakistan (trauma (31.5%) and topical steroid (6.6%). In a French setting, Dethorey et al. [18] identified risk factors for IK as contact lens use (48.1%) and ocular surface diseases (33.7%). 

In our study, ulcer size was moderate in 41.1% of patients and severe in 40.2% of patients. At the same time 54.2% of mild depth, 24.3% moderate depth, and 21.5% severe depth ulcers were seen. In the Agarwal et al. study, among the keratitis patients enrolled, 13.9% experienced thinning greater than 60% or developed a descemetocele, while 3.4% had perforations [19]. In Darren Shu Jeng Ting et al.’s study, a small epithelial defect-size ulcer in 60.8% was noted [20].

In our study on gram staining, *Staphylococcus epidermidis* was the most common gram-positive cocci isolated (3.7%), followed by *Staphylococcus aureus* (2.8%). *Pseudomonas aeruginosa* (1.86%) was the only gram-negative bacteria isolated, and in 58% of patients, fungal elements were seen on KOH mount. On culture, fungal organisms were the most common isolate (57.9%), followed by bacterial (12.1%), and no growth was seen on culture in 30% of cases. One patient had both bacterial and fungal isolates positive. *Staphylococcus epidermidis* (3.7%) was the most common organism among the bacterial isolates on culture. And Fusarium species (40%) was the most common fungal isolate, followed by *Aspergillus flavus* and *Niger* (7.5%) each.

In the Indian setting of the Kaliamurthy et al. [21] study, done in southern India, *S. epidermis* (44.0%), *S. aureus* (19.5%), and *S. pneumonia* (11.6%) were found. Rautaraya et al. study, which was done in a tertiary care center in eastern India [22], observed *Aspergillus spp.* (23.1%); *Fusarium spp.* (19.2%); and *Staphylococci* (5.4%) as the most common microorganisms. Lin et al. [23] study in southern India observed *Fusarium spp.* (15.5%); *S. pneumoniae* (7.3%); and *Pseudomonas* (5.0%) as agents. In Lalitha et al. [24] study done in southern India, *Fusarium spp.* (14.5%), *Aspergillus* species (8.8%), and *S. pneumoniae* (7%) were the most common organisms. In most Indian studies, fungal agents were more predominantly the factors attributed to microbiological agents, similar to our study findings. In the Ting et al. [5] study from Nottingham, UK, *Pseudomonas* (23.6%), *S. aureus* (15.9%), and *Streptococci* (13.5%) were observed as microbiological agents among IK patients. Coagulase negative *Staphylococci* (CoNS) (33.9%), *S. aureus* (11.2%), and *Pseudomonas spp. *(17.7%) were documented microbiological agents in the Green et al. [11] study in Queensland, Australia. In UK studies, Kaye et al. [25] observed CoNS (26.3%), *Enterobacteriaceae* (15.3%), and *Streptococci* (13.9%). In another Indian study conducted by Acharya et al. in northern India [26] in 2015-2017, among the 1169 study population, the agents observed were CoNS (46.3%), *Pseudomonas* species. (16.2%), and *Streptococci* (15.5%). 

In our study, among the 93 of 107 patients who followed up, 51 (54.8%) underwent medical and 42 (45.16%) underwent surgical management. Most patients with surgical management with TPK had fungal isolates (83%) positive, and 41.2% of medically treated patients also had fungal isolates positive. Of the medically managed patients, 49 of 51 (96%) had healed ulcers by two months; in one patient the ulcer was not healed, and one patient had descemetocele. Among the 96% healed ulcers, 72.5% ulcers were healed by nebulomacular opacity, and 23.5% ulcers healed by leucomatous opacity. Half of the mild-size ulcers were healed by 15 days the remaining half by the one-month follow-up, almost all moderate-size ulcers by two months, and severe-size ulcers by the two-month follow-up visit. Among the surgically managed patients at the six-month follow-up visit, the graft was found to be in place in most of the patients (95.2%), while 2.4% each showed complications of graft rejection and endophthalmitis. In our study, all (100%) severe-depth ulcers required TPK, and 73% of moderate-depth ulcers required TPK.

In a study conducted by Rogers et al. [27], 98.6% were cured; among these, 56% were treated by medical therapy, and 44% with therapeutic keratoplasty. However, the study documented failed drafts and a second TPK was done for a few patients. After TPK, 53.1% maintained clear grafts. In a study by Ting DSJ et al. [20], surgical intervention was done in 16.3% of patients, while the majority (84%) of the cases were healed by medical treatment. Complications like corneal perforation in 8.8% and enucleation/evisceration in 1.4% were seen. Delayed healing of ulcers (>30 days from initial presentation) and poor visual outcome were seen most commonly in ages >50 years, with reduced presenting vision. In a study by B. Rautaraya et al. [28], healed scar tissue was seen as a clinical outcome in 35.6% of mycotic keratitis cases. TPK was done in 19.7% of cases, 3.4% had evisceration, and 18.9% for impending perforation had application of glue with bandage contact lens. 6.1% were unchanged, and 16.3% were lost to follow-up. A large number of patients who presented late (> 10 days) had poor prognoses, like penetrating keratoplasty (75.9%) and bandage contact lenses in 60%. In another study conducted by Usha Gopinathan et al. [29], a large number of patients (50.8%) with fungal keratitis required surgical intervention as compared to 43.2% in BK and 17.4% in *Acanthamoeba keratitis*. 

Limitations

Our study is a single-centric study; hence, the results obtained cannot be generalized to all regions in India. Previous medical management given to patients elsewhere was not taken into consideration. And since our study is follow-up-based, due to more frequent follow-up visits, lost follow-up was seen in our study.

## Conclusions

We conclude that *Staphylococcus epidermidis* and *Staphylococcus aureus* were the most common bacterial agents isolated on gram staining and *Pseudomonas aeruginosa* was the only gram-negative bacillus isolated on gram staining. Fungal isolates were the most common isolates observed on culture, seen in more than half of the infectious keratitis patients in our study. Among them, Fusarium species were major attributes. Around one-third had no growth in culture.

In our study, history of ocular trauma and diabetes were major risk factors. In the majority of patients with medical management, the ulcer was healed in two months. Surgical management with TPK was required in severely deep ulcers. Fungal isolates were positive in the majority of surgically managed patients. We recommend further research be conducted in a generalized population, including urban settings, to understand the risk factors, microbiological profile, and treatment outcomes for infectious keratitis.
